# Bis[4-(4-methoxy­phen­yl)-4*H*-1,2,4-triazole-κ*N* ^1^]bis­(thio­cyanato-κ*N*)zinc(II)

**DOI:** 10.1107/S1600536808008325

**Published:** 2008-04-02

**Authors:** Ya Zuo

**Affiliations:** a College of Science, Inner Mongolia Agricultural University, Inner Mongolia 010018, People’s Republic of China

## Abstract

In the title complex, [Zn(NCS)_2_(C_9_H_9_N_3_O)_2_], the Zn^II^ ion is coordinated by two N atoms from the NCS^−^ anions and two N atoms from two 4-(4-methoxy­phen­yl)-4*H*-1,2,4-triazole ligands in a slightly distorted tetra­hedral geometry. Three inter­molecular weak hydrogen-bonding contacts of the types C—H⋯N, C—H⋯S and C—H⋯O are observed in the crystal structure.

## Related literature

For related literature, see: Han *et al.* (2006 ▶); Ling & Zhang (2007 ▶); Thomann *et al.* (1994 ▶); Yin *et al.* (2007 ▶); Zhao *et al.* (2002 ▶); Zhou *et al.* (2007 ▶).
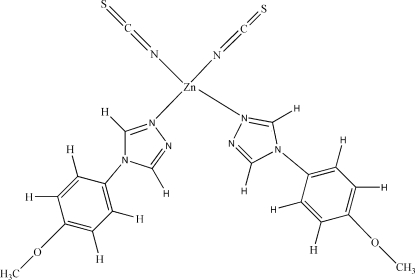


## Experimental

### 

#### Crystal data

[Zn(NCS)_2_(C_9_H_9_N_3_O)_2_]
*M* *_r_* = 531.91
Monoclinic, 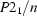

*a* = 7.812 (3) Å
*b* = 17.111 (6) Å
*c* = 18.201 (6) Å
β = 99.726 (6)°
*V* = 2398.0 (15) Å^3^
*Z* = 4
Mo *K*α radiation
μ = 1.23 mm^−1^
*T* = 293 (2) K
0.16 × 0.12 × 0.10 mm

#### Data collection

Bruker SMART CCD area-detector diffractometer
Absorption correction: multi-scan (*SADABS*; Sheldrick, 1996 ▶) *T*_min_ = 0.700, *T*_max_ = 0.884
13731 measured reflections
4932 independent reflections
2127 reflections with *I* > 2σ(*I*)
*R*_int_ = 0.121

#### Refinement

*R*[*F* ^2^ > 2σ(*F* ^2^)] = 0.067
*wR*(*F* ^2^) = 0.158
*S* = 0.97
4932 reflections
300 parameters
H-atom parameters constrained
Δρ_max_ = 0.39 e Å^−3^
Δρ_min_ = −0.33 e Å^−3^

### 

Data collection: *SMART* (Bruker, 1998 ▶); cell refinement: *SAINT* (Bruker, 1999 ▶); data reduction: *SAINT*; program(s) used to solve structure: *SHELXS97* (Sheldrick, 2008 ▶); program(s) used to refine structure: *SHELXL97* (Sheldrick, 2008 ▶); molecular graphics: *SHELXTL* (Sheldrick, 2008 ▶); software used to prepare material for publication: *SHELXTL*.

## Supplementary Material

Crystal structure: contains datablocks I, global. DOI: 10.1107/S1600536808008325/si2077sup1.cif

Structure factors: contains datablocks I. DOI: 10.1107/S1600536808008325/si2077Isup2.hkl

Additional supplementary materials:  crystallographic information; 3D view; checkCIF report

## Figures and Tables

**Table:** 

Zn1—N8	1.923 (6)
Zn1—N7	1.970 (6)
Zn1—N1	2.005 (5)
Zn1—N4	2.009 (5)

**Table:** 

N8—Zn1—N7	112.9 (2)
N8—Zn1—N1	112.1 (2)
N7—Zn1—N1	108.5 (2)
N8—Zn1—N4	116.4 (2)
N7—Zn1—N4	103.8 (2)
N1—Zn1—N4	102.2 (2)

**Table 2:** Hydrogen-bond geometry (Å, °)

*D*—H⋯*A*	*D*—H	H⋯*A*	*D*⋯*A*	*D*—H⋯*A*
C1—H1⋯N5^i^	0.93	2.51	3.438 (8)	177
C7—H7⋯S1^ii^	0.93	2.86	3.735 (6)	158
C10—H10⋯O1^iii^	0.93	2.42	3.265 (8)	151

Symmetry codes: (i) 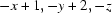; (ii) 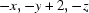; (iii) 
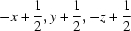
.

**Fig. 1.**
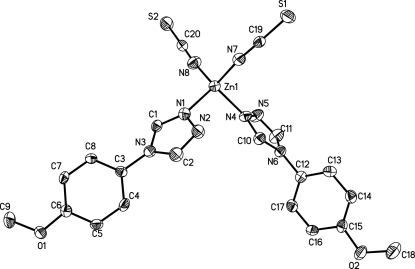
The ORTEP plot of the title complex, showing 30% probability displacement ellipsoids and the atom-numbering schemes. H atoms have been omitted for clarity.

## supplementary crystallographic information

### Comment

The organic ligands, 1,2,4-triazole and its derivatives continue to attract considerable attention due to the luminescent, magnetic, and electronic properties of their complexes and the potential application in material science (Yin *et al.*, 2007; Zhou, *et al.*, 2007; Han *et al.*, 2006). On the other hand, 1,2,4-triazole and its derivatives combine the coordination geometry of both pyrazole and imidazole with regard to the arrangement and coordination of their three heteroatoms. Compared with the chelating to the metal centers with the nitrogen atom in the 1,2-positions (Thomann *et al.*, 1994), it is familiar that the nitrogen atom serve as the solo coordinated atom (Ling & Zhang, 2007). In order to explore furthur the coordination chemistry of the 4-(4-methoxyphenyl)-4H-1,2,4-triazole system (hereafter abbreviated as *L*), the title complex was synthesized.

The coordination geometry of the Zn^II^ ion is a slightly distorted tetrahedron, in which each Zn^II^ ion is coordinated to two nitrogen atoms from the NCS^-^ anions and two nitrogen atoms from two ligands *L*. (Fig. 1). The separations of Zn—N range from 1.923 (6) to 2.009 (5)Å (Table 1), which is close to the corresponding distances in the 4-coordinated Zn compound (2.050 (4)Å and 2.023 (4) Å) reported previously (Han *et al.*, 2006), while the average distance of Zn—N is 2.162 (2)Å in the 6-coordinated (Ling & Zhang, 2007) and 2.065 (2)Å in the 5-coordinated (Yin *et al.*, 2007) compounds, respectively. The bond angles around Zn^II^ ions vary between 102.2 (2)° and 116.4 (2)°. The central Zn^II^ ion, is coordinated with the *L* ligands in the 1-position, which is the common coordination mode (Ling & Zhang, 2007) of 1,2,4-Triazole and its derivatives, differently from that in the 1,2-position (Thomann *et al.*, 1994). As the important structural parameters, the dihedral angles between the triazole and phenyl rings of the two *L* ligands are distinct, that of C1—N3—C3—C8 is equal to 39.2 (9)°, while the value of C10—N6—C12—C17 is 93.8 (8)°. With regard to the two *trans* thiocyanato NCS^-^ anions, the group is almost linear with an N7—C19—S1 angle of 178.8 (8)° and N8—C20—S2 angle of 178.1 (7)°. The connection between Zn atoms and NCS groups are bent with a C20—N8—Zn1 angle of 168.1 (6)°, and a C19—N7—Zn1 angle of 160.9 (6)° (Zhao *et al.*, 2002). Three intermoleular hydrogen bonding contacts of the type C—H···N, C—H···S and C—H···O are observed in the title structure (Table 2).

### Experimental

The compound was synthesized under hydrothermal conditions. A mixture of *L* (*L*=4-(1,2,4-triazol)-1-methoxy-benzene) (0.3 mmol, 0.045 g), ZnSO_4_˙7H_2_O (0.1 mmol, 0.029 g), KSCN (0.2 mmol, 0.019 g) and water (10 mL) was placed in a 25 mL acid digestion bomb and heated at 160° for two days, then equably cooled to room temperature for three days. After washed by 5 ml water for twice, colorless block crystals of the title compound were obtained.

### Refinement

The H atoms (methyl) on the ligands were allowed to ride on their parent atoms with C—H distances of 0.96Å and *U*_iso_(H)=1.5*U*_eq_(C), and the rest C—H distances of 0.93Å and *U*_iso_(H)=1.2*U*_eq_(C). All of the non-hydrogen atoms were refined anisotropically.

### Crystal data

**Table:**

[Zn(NCS)_2_(C_9_H_9_N_3_O)_2_]	*F*_000_ = 1088
*M**_r_* = 531.91	*D*_x_ = 1.473 Mg m^−^^3^
Monoclinic, *P*2_1_/*n*	Mo *K*α radiation λ = 0.71073 Å
*a* = 7.812 (3) Å	Cell parameters from 713 reflections
*b* = 17.111 (6) Å	θ = 2.6–21.1º
*c* = 18.201 (6) Å	µ = 1.23 mm^−^^1^
β = 99.726 (6)º	*T* = 293 (2) K
*V* = 2398.0 (15) Å^3^	Block, colorless
*Z* = 4	0.16 × 0.12 × 0.10 mm

### Data collection

**Table:**

Bruker SMART CCD area-detector diffractometer	4932 independent reflections
Radiation source: fine-focus sealed tube	2127 reflections with *I* > 2σ(*I*)
Monochromator: graphite	*R*_int_ = 0.121
*T* = 293(2) K	θ_max_ = 26.5º
φ and O scans	θ_min_ = 1.6º
Absorption correction: multi-scan(SADABS; Sheldrick, 1996)	*h* = −9→9
*T*_min_ = 0.700, *T*_max_ = 0.884	*k* = −21→14
13731 measured reflections	*l* = −22→18

### Refinement

**Table:**

Refinement on *F*^2^	Secondary atom site location: difference Fourier map
Least-squares matrix: full	Hydrogen site location: inferred from neighbouring sites
*R*[*F*^2^ > 2σ(*F*^2^)] = 0.067	H-atom parameters constrained
*wR*(*F*^2^) = 0.158	*w* = 1/[σ^2^(*F*_o_^2^) + (0.0555*P*)^2^] where *P* = (*F*_o_^2^ + 2*F*_c_^2^)/3
*S* = 0.97	(Δ/σ)_max_ = 0.001
4932 reflections	Δρ_max_ = 0.39 e Å^−^^3^
300 parameters	Δρ_min_ = −0.33 e Å^−^^3^
Primary atom site location: structure-invariant direct methods	Extinction correction: none

### Special details

**Table:**

Geometry. All e.s.d.'s (except the e.s.d. in the dihedral angle between two l.s. planes) are estimated using the full covariance matrix. The cell e.s.d.'s are taken into account individually in the estimation of e.s.d.'s in distances, angles and torsion angles; correlations between e.s.d.'s in cell parameters are only used when they are defined by crystal symmetry. An approximate (isotropic) treatment of cell e.s.d.'s is used for estimating e.s.d.'s involving l.s. planes.
Refinement. Refinement of *F*^2^ against ALL reflections. The weighted *R*-factor *wR* and goodness of fit *S* are based on *F*^2^, conventional *R*-factors *R* are based on *F*, with *F* set to zero for negative *F*^2^. The threshold expression of *F*^2^ > σ(*F*^2^) is used only for calculating *R*-factors(gt) *etc*. and is not relevant to the choice of reflections for refinement. *R*-factors based on *F*^2^ are statistically about twice as large as those based on *F*, and *R*- factors based on ALL data will be even larger.

### Fractional atomic coordinates and isotropic or equivalent isotropic displacement parameters (Å^2^)

**Table:**

	*x*	*y*	*z*	*U*_iso_*/*U*_eq_	
Zn1	0.36832 (9)	1.10583 (5)	0.05909 (4)	0.0434 (3)	
N1	0.2576 (6)	1.0131 (3)	0.0996 (3)	0.0448 (14)	
N2	0.2447 (7)	1.0191 (4)	0.1742 (3)	0.0506 (15)	
N3	0.1960 (6)	0.8971 (4)	0.1360 (3)	0.0417 (13)	
O1	0.0331 (6)	0.5821 (3)	0.1525 (2)	0.0495 (12)	
C1	0.2292 (7)	0.9405 (4)	0.0785 (3)	0.0393 (16)	
H1	0.2317	0.9216	0.0307	0.047*	
C2	0.2083 (9)	0.9483 (5)	0.1939 (4)	0.055 (2)	
H2	0.1927	0.9346	0.2418	0.066*	
C3	0.1559 (8)	0.8147 (4)	0.1374 (3)	0.0364 (15)	
C4	0.2212 (8)	0.7714 (4)	0.1995 (3)	0.0436 (17)	
H4	0.2949	0.7943	0.2391	0.052*	
C5	0.1762 (8)	0.6938 (4)	0.2023 (3)	0.0429 (17)	
H5	0.2179	0.6646	0.2447	0.051*	
C6	0.0701 (8)	0.6587 (4)	0.1433 (3)	0.0383 (16)	
C7	0.0088 (8)	0.7023 (4)	0.0807 (3)	0.0462 (18)	
H7	−0.0610	0.6791	0.0400	0.055*	
C8	0.0506 (8)	0.7795 (4)	0.0784 (3)	0.0467 (18)	
H8	0.0073	0.8089	0.0364	0.056*	
C9	−0.0628 (8)	0.5413 (4)	0.0903 (4)	0.060 (2)	
H9A	−0.1752	0.5649	0.0767	0.090*	
H9B	−0.0762	0.4876	0.1035	0.090*	
H9C	−0.0014	0.5440	0.0489	0.090*	
N4	0.6037 (6)	1.1085 (3)	0.1237 (3)	0.0423 (13)	
N5	0.7566 (7)	1.1233 (4)	0.0996 (3)	0.0586 (18)	
N6	0.8049 (6)	1.1152 (3)	0.2222 (3)	0.0421 (14)	
O2	1.1582 (6)	1.1055 (3)	0.5135 (2)	0.0655 (14)	
C10	0.6367 (8)	1.1026 (4)	0.1966 (3)	0.0464 (16)	
H10	0.5541	1.0912	0.2264	0.056*	
C11	0.8730 (9)	1.1266 (5)	0.1594 (4)	0.065 (2)	
H11	0.9902	1.1359	0.1592	0.078*	
C12	0.8924 (7)	1.1139 (4)	0.2991 (3)	0.0385 (15)	
C13	0.8962 (8)	1.1802 (4)	0.3406 (3)	0.0474 (18)	
H13	0.8410	1.2253	0.3202	0.057*	
C14	0.9835 (8)	1.1797 (4)	0.4138 (4)	0.0509 (18)	
H14	0.9860	1.2246	0.4429	0.061*	
C15	1.0660 (7)	1.1130 (4)	0.4433 (3)	0.0436 (16)	
C16	1.0579 (8)	1.0461 (4)	0.4010 (4)	0.0505 (18)	
H16	1.1114	1.0007	0.4214	0.061*	
C17	0.9708 (8)	1.0461 (4)	0.3285 (4)	0.0462 (18)	
H17	0.9651	1.0009	0.2999	0.055*	
C18	1.1740 (10)	1.1722 (5)	0.5603 (4)	0.080 (3)	
H18A	1.2279	1.2138	0.5372	0.120*	
H18B	1.2440	1.1595	0.6074	0.120*	
H18C	1.0608	1.1884	0.5681	0.120*	
S1	0.1894 (3)	1.35631 (13)	0.11268 (10)	0.0765 (7)	
N7	0.2543 (7)	1.2013 (4)	0.0874 (3)	0.0527 (16)	
C19	0.2283 (9)	1.2663 (5)	0.0986 (3)	0.0445 (18)	
S2	0.3020 (3)	1.10216 (14)	−0.19983 (10)	0.0796 (7)	
N8	0.3645 (7)	1.0978 (4)	−0.0466 (3)	0.0619 (17)	
C20	0.3357 (8)	1.1003 (4)	−0.1112 (4)	0.0500 (17)	

### Atomic displacement parameters (Å^2^)

**Table:**

	*U*^11^	*U*^22^	*U*^33^	*U*^12^	*U*^13^	*U*^23^
Zn1	0.0453 (4)	0.0419 (5)	0.0414 (4)	0.0001 (5)	0.0025 (3)	0.0030 (4)
N1	0.045 (3)	0.045 (4)	0.045 (3)	−0.001 (3)	0.007 (3)	−0.003 (3)
N2	0.070 (4)	0.047 (4)	0.036 (3)	−0.003 (3)	0.012 (3)	−0.007 (3)
N3	0.051 (3)	0.045 (4)	0.029 (3)	−0.001 (3)	0.008 (2)	−0.001 (3)
O1	0.062 (3)	0.040 (3)	0.046 (3)	−0.008 (2)	0.006 (2)	0.001 (2)
C1	0.041 (4)	0.043 (5)	0.033 (4)	0.000 (3)	0.006 (3)	−0.009 (3)
C2	0.073 (5)	0.055 (6)	0.039 (4)	−0.002 (4)	0.013 (4)	−0.005 (4)
C3	0.041 (4)	0.032 (4)	0.037 (4)	0.005 (3)	0.010 (3)	0.000 (3)
C4	0.047 (4)	0.051 (5)	0.031 (4)	−0.007 (4)	0.000 (3)	−0.009 (3)
C5	0.044 (4)	0.048 (5)	0.036 (4)	0.000 (3)	0.004 (3)	0.011 (3)
C6	0.033 (3)	0.043 (5)	0.041 (4)	−0.002 (3)	0.011 (3)	−0.002 (3)
C7	0.052 (4)	0.047 (5)	0.035 (4)	−0.014 (4)	−0.007 (3)	−0.007 (3)
C8	0.059 (4)	0.043 (5)	0.033 (4)	−0.009 (4)	−0.004 (3)	0.012 (3)
C9	0.061 (5)	0.050 (5)	0.068 (5)	−0.010 (4)	0.006 (4)	−0.019 (4)
N4	0.037 (3)	0.052 (4)	0.039 (3)	0.003 (3)	0.007 (2)	0.008 (3)
N5	0.046 (3)	0.087 (5)	0.043 (3)	−0.005 (3)	0.009 (3)	0.009 (3)
N6	0.037 (3)	0.049 (4)	0.039 (3)	−0.003 (3)	0.006 (3)	0.000 (3)
O2	0.071 (3)	0.070 (4)	0.048 (3)	0.003 (3)	−0.012 (2)	−0.011 (3)
C10	0.042 (4)	0.052 (5)	0.045 (4)	−0.002 (4)	0.007 (3)	0.000 (4)
C11	0.045 (4)	0.099 (7)	0.052 (5)	−0.012 (4)	0.012 (4)	0.006 (5)
C12	0.033 (3)	0.046 (5)	0.039 (4)	0.001 (3)	0.009 (3)	−0.003 (4)
C13	0.048 (4)	0.048 (5)	0.046 (4)	0.008 (4)	0.008 (4)	0.008 (4)
C14	0.058 (5)	0.041 (5)	0.053 (4)	0.003 (4)	0.006 (4)	−0.009 (4)
C15	0.039 (3)	0.049 (5)	0.043 (4)	−0.009 (4)	0.006 (3)	−0.006 (4)
C16	0.057 (4)	0.040 (5)	0.050 (4)	0.013 (4)	−0.004 (4)	0.006 (4)
C17	0.045 (4)	0.040 (5)	0.053 (5)	0.009 (4)	0.008 (3)	−0.011 (4)
C18	0.072 (5)	0.100 (8)	0.059 (5)	0.011 (5)	−0.010 (4)	−0.018 (5)
S1	0.128 (2)	0.0453 (13)	0.0492 (12)	0.0171 (13)	−0.0041 (12)	−0.0014 (10)
N7	0.060 (4)	0.050 (4)	0.045 (3)	0.003 (3)	−0.001 (3)	−0.001 (3)
C19	0.053 (4)	0.048 (5)	0.030 (4)	−0.005 (4)	0.000 (3)	0.005 (4)
S2	0.1267 (18)	0.0637 (15)	0.0433 (11)	−0.0087 (14)	−0.0001 (12)	−0.0048 (11)
N8	0.065 (4)	0.070 (5)	0.048 (4)	0.004 (4)	0.003 (3)	0.001 (4)
C20	0.054 (4)	0.038 (4)	0.055 (5)	0.001 (4)	0.001 (4)	−0.001 (4)

### Geometric parameters (Å, °)

**Table:**

Zn1—N8	1.923 (6)	N4—C10	1.312 (7)
Zn1—N7	1.970 (6)	N4—N5	1.365 (6)
Zn1—N1	2.005 (5)	N5—C11	1.296 (7)
Zn1—N4	2.009 (5)	N6—C10	1.336 (7)
N1—C1	1.309 (8)	N6—C11	1.354 (7)
N1—N2	1.382 (7)	N6—C12	1.450 (7)
N2—C2	1.308 (8)	O2—C15	1.363 (7)
N3—C1	1.344 (7)	O2—C18	1.417 (8)
N3—C2	1.361 (8)	C10—H10	0.9300
N3—C3	1.445 (8)	C11—H11	0.9300
O1—C6	1.359 (7)	C12—C13	1.360 (9)
O1—C9	1.430 (7)	C12—C17	1.377 (8)
C1—H1	0.9300	C13—C14	1.391 (8)
C2—H2	0.9300	C13—H13	0.9300
C3—C8	1.375 (8)	C14—C15	1.374 (9)
C3—C4	1.376 (8)	C14—H14	0.9300
C4—C5	1.378 (9)	C15—C16	1.375 (9)
C4—H4	0.9300	C16—C17	1.379 (8)
C5—C6	1.379 (8)	C16—H16	0.9300
C5—H5	0.9300	C17—H17	0.9300
C6—C7	1.379 (8)	C18—H18A	0.9600
C7—C8	1.364 (9)	C18—H18B	0.9600
C7—H7	0.9300	C18—H18C	0.9600
C8—H8	0.9300	S1—C19	1.598 (8)
C9—H9A	0.9600	N7—C19	1.155 (8)
C9—H9B	0.9600	S2—C20	1.591 (7)
C9—H9C	0.9600	N8—C20	1.160 (7)
			
N8—Zn1—N7	112.9 (2)	H9B—C9—H9C	109.5
N8—Zn1—N1	112.1 (2)	C10—N4—N5	107.9 (5)
N7—Zn1—N1	108.5 (2)	C10—N4—Zn1	126.4 (4)
N8—Zn1—N4	116.4 (2)	N5—N4—Zn1	125.5 (4)
N7—Zn1—N4	103.8 (2)	C11—N5—N4	105.4 (5)
N1—Zn1—N4	102.2 (2)	C10—N6—C11	103.4 (5)
C1—N1—N2	108.8 (5)	C10—N6—C12	127.6 (5)
C1—N1—Zn1	134.9 (5)	C11—N6—C12	129.0 (5)
N2—N1—Zn1	114.2 (4)	C15—O2—C18	117.8 (6)
C2—N2—N1	104.9 (5)	N4—C10—N6	110.7 (6)
C1—N3—C2	104.5 (6)	N4—C10—H10	124.6
C1—N3—C3	128.5 (5)	N6—C10—H10	124.6
C2—N3—C3	127.0 (6)	N5—C11—N6	112.6 (6)
C6—O1—C9	117.9 (5)	N5—C11—H11	123.7
N1—C1—N3	109.9 (6)	N6—C11—H11	123.7
N1—C1—H1	125.0	C13—C12—C17	121.3 (6)
N3—C1—H1	125.0	C13—C12—N6	119.1 (6)
N2—C2—N3	111.9 (6)	C17—C12—N6	119.7 (6)
N2—C2—H2	124.0	C12—C13—C14	119.3 (6)
N3—C2—H2	124.0	C12—C13—H13	120.4
C8—C3—C4	119.7 (6)	C14—C13—H13	120.4
C8—C3—N3	121.1 (6)	C15—C14—C13	120.0 (6)
C4—C3—N3	119.2 (6)	C15—C14—H14	120.0
C3—C4—C5	119.2 (6)	C13—C14—H14	120.0
C3—C4—H4	120.4	O2—C15—C14	125.7 (7)
C5—C4—H4	120.4	O2—C15—C16	114.3 (7)
C4—C5—C6	121.0 (6)	C14—C15—C16	120.0 (6)
C4—C5—H5	119.5	C15—C16—C17	120.2 (6)
C6—C5—H5	119.5	C15—C16—H16	119.9
O1—C6—C7	125.0 (6)	C17—C16—H16	119.9
O1—C6—C5	115.9 (6)	C12—C17—C16	119.2 (6)
C7—C6—C5	119.2 (6)	C12—C17—H17	120.4
C8—C7—C6	119.9 (6)	C16—C17—H17	120.4
C8—C7—H7	120.1	O2—C18—H18A	109.5
C6—C7—H7	120.1	O2—C18—H18B	109.5
C7—C8—C3	121.0 (6)	H18A—C18—H18B	109.5
C7—C8—H8	119.5	O2—C18—H18C	109.5
C3—C8—H8	119.5	H18A—C18—H18C	109.5
O1—C9—H9A	109.5	H18B—C18—H18C	109.5
O1—C9—H9B	109.5	C19—N7—Zn1	161.0 (6)
H9A—C9—H9B	109.5	N7—C19—S1	178.8 (7)
O1—C9—H9C	109.5	C20—N8—Zn1	168.1 (6)
H9A—C9—H9C	109.5	N8—C20—S2	178.1 (7)
			
N8—Zn1—N1—C1	24.0 (7)	N7—Zn1—N4—N5	−108.5 (5)
N7—Zn1—N1—C1	149.4 (6)	N1—Zn1—N4—N5	138.7 (5)
N4—Zn1—N1—C1	−101.4 (6)	C10—N4—N5—C11	−0.9 (8)
N8—Zn1—N1—N2	−175.1 (4)	Zn1—N4—N5—C11	174.2 (5)
N7—Zn1—N1—N2	−49.7 (4)	N5—N4—C10—N6	1.8 (8)
N4—Zn1—N1—N2	59.5 (4)	Zn1—N4—C10—N6	−173.2 (4)
C1—N1—N2—C2	−0.1 (7)	C11—N6—C10—N4	−1.9 (8)
Zn1—N1—N2—C2	−165.9 (4)	C12—N6—C10—N4	−180.0 (6)
N2—N1—C1—N3	0.5 (7)	N4—N5—C11—N6	−0.3 (9)
Zn1—N1—C1—N3	162.2 (4)	C10—N6—C11—N5	1.4 (9)
C2—N3—C1—N1	−0.7 (7)	C12—N6—C11—N5	179.4 (6)
C3—N3—C1—N1	179.2 (5)	C10—N6—C12—C13	−86.6 (8)
N1—N2—C2—N3	−0.4 (7)	C11—N6—C12—C13	95.8 (8)
C1—N3—C2—N2	0.7 (7)	C10—N6—C12—C17	93.7 (8)
C3—N3—C2—N2	−179.3 (6)	C11—N6—C12—C17	−83.8 (9)
C1—N3—C3—C8	−39.2 (9)	C17—C12—C13—C14	1.1 (10)
C2—N3—C3—C8	140.7 (6)	N6—C12—C13—C14	−178.5 (5)
C1—N3—C3—C4	142.3 (6)	C12—C13—C14—C15	0.6 (10)
C2—N3—C3—C4	−37.7 (9)	C18—O2—C15—C14	−1.2 (9)
C8—C3—C4—C5	−1.6 (9)	C18—O2—C15—C16	179.4 (6)
N3—C3—C4—C5	176.9 (5)	C13—C14—C15—O2	178.7 (6)
C3—C4—C5—C6	1.4 (9)	C13—C14—C15—C16	−1.9 (10)
C9—O1—C6—C7	6.3 (9)	O2—C15—C16—C17	−179.0 (6)
C9—O1—C6—C5	−174.3 (5)	C14—C15—C16—C17	1.6 (10)
C4—C5—C6—O1	−179.4 (5)	C13—C12—C17—C16	−1.4 (10)
C4—C5—C6—C7	0.1 (9)	N6—C12—C17—C16	178.2 (5)
O1—C6—C7—C8	178.2 (6)	C15—C16—C17—C12	0.1 (10)
C5—C6—C7—C8	−1.3 (9)	N8—Zn1—N7—C19	−71.6 (17)
C6—C7—C8—C3	1.1 (10)	N1—Zn1—N7—C19	163.5 (16)
C4—C3—C8—C7	0.4 (10)	N4—Zn1—N7—C19	55.3 (17)
N3—C3—C8—C7	−178.0 (6)	Zn1—N7—C19—S1	105 (36)
N8—Zn1—N4—C10	−169.6 (6)	N7—Zn1—N8—C20	−29 (3)
N7—Zn1—N4—C10	65.7 (6)	N1—Zn1—N8—C20	93 (3)
N1—Zn1—N4—C10	−47.1 (6)	N4—Zn1—N8—C20	−149 (3)
N8—Zn1—N4—N5	16.2 (6)	Zn1—N8—C20—S2	180 (100)

### Hydrogen-bond geometry (Å, °)

**Table:**

*D*—H···*A*	*D*—H	H···*A*	*D*···*A*	*D*—H···*A*
C1—H1···N5^i^	0.93	2.51	3.438 (8)	177
C7—H7···S1^ii^	0.93	2.86	3.735 (6)	158
C10—H10···O1^iii^	0.93	2.42	3.265 (8)	151

Symmetry codes: (i) −*x*+1, −*y*+2, −*z*; (ii) −*x*, −*y*+2, −*z*; (iii) −*x*+1/2, *y*+1/2, −*z*+1/2.
